# Application of machine learning approaches in predicting clinical outcomes in older adults – a systematic review and meta-analysis

**DOI:** 10.1186/s12877-023-04246-w

**Published:** 2023-09-14

**Authors:** Robert T. Olender, Sandipan Roy, Prasad S. Nishtala

**Affiliations:** 1 Department of Life Sciences, University of Bath, Bath, BA2 7AY UK; 2 Department of Mathematical Sciences, University of Bath, Bath, BA2 7AY UK; 3 Department of Life Sciences & Centre for Therapeutic Innovation, University of Bath, Bath, BA2 7AY UK

**Keywords:** Older adults, Machine learning, Predictive modelling, Model performance evaluation, Health informatics, Risk management

## Abstract

**Background:**

Machine learning-based prediction models have the potential to have a considerable positive impact on geriatric care.

**Design:**

Systematic review and meta-analyses.

**Participants:**

Older adults (≥ 65 years) in any setting.

**Intervention:**

Machine learning models for predicting clinical outcomes in older adults were evaluated. A random-effects meta-analysis was conducted in two grouped cohorts, where the predictive models were compared based on their performance in predicting mortality i) under and including 6 months ii) over 6 months.

**Outcome measures:**

Studies were grouped into two groups by the clinical outcome, and the models were compared based on the area under the receiver operating characteristic curve metric.

**Results:**

Thirty-seven studies that satisfied the systematic review criteria were appraised, and eight studies predicting a mortality outcome were included in the meta-analyses**.** We could only pool studies by mortality as there were inconsistent definitions and sparse data to pool studies for other clinical outcomes. The area under the receiver operating characteristic curve from the meta-analysis yielded a summary estimate of 0.80 (95% CI: 0.76 – 0.84) for mortality within 6 months and 0.81 (95% CI: 0.76 – 0.86) for mortality over 6 months, signifying good discriminatory power.

**Conclusion:**

The meta-analysis indicates that machine learning models display good discriminatory power in predicting mortality. However, more large-scale validation studies are necessary. As electronic healthcare databases grow larger and more comprehensive, the available computational power increases and machine learning models become more sophisticated; there should be an effort to integrate these models into a larger research setting to predict various clinical outcomes.

**Supplementary Information:**

The online version contains supplementary material available at 10.1186/s12877-023-04246-w.

## Background

Older adults aged ≥ 65 years are the highest healthcare consumers, accounting for the largest and disproportionate share of hospitalisations and in-hospital deaths [1]. Moreover, this population demonstrates the highest prevalence of multimorbidity [2], emphasizing the importance of optimised clinical care and healthcare resource allocation.Traditional statistical methods for predicting clinical outcomes in this age group have limitations due to their parsimonious nature and predetermined modelling assumptions. Conversely, machine learning, which employs algorithms to analyse extensive datasets and generate predictions based on pre-set criteria, does not share these drawbacks [3].

The application of machine learning in geriatric medicine is already evidentin multiple clinical areas such as cancer diagnosis [4], predicting falls [5], mortality [6], and other therapeutic areas [7–10]. However, these applications predominantly underwent internal validation, with limited exposure in real-world clinical settings. Some recent exceptions that applied machine learning models validated in external clinical cohorts are in predicting real-time emergency department visits [11], pain perception in older adults with cognitive impairment [12] and the risk of myeloid neoplasms [13].

Despite the aforementioned progress, a comprehensive overview that synthesis these findings is lacking. To the authors' knowledge, this is the first systematic literature review and meta-analysis focused on the use of machine learning to predict clinical outcomes in the older adult demographic (≥ 65 years). While a previous review addressed machine learning in geriatric care for chronic diseases, it lacked the depth and did not include a meta-analysis [14]. Existing studies often emphasise diagnostic applications, yet literature that predicts relevant clinical outcomes those that can directly enhance geriatric care and influence prescribing policies is limited.

The potential applications of machine learning in geriatrics are vast. It could transform care by forecasting frailty [15] enabling proactive clinical interventions or adjusting treatment courses. For example, heart failure risk predictions might guide clinical decisions to mitigate adverse outcomes in this frail population. Notably, falls are a significant concern, with around a third of those aged > 65 years experiencing at least one accidental fall annually, resulting in injuries for 20% and hospitalisation for 5% [16]. Early identification of high-risk individuals could trigger preventive strategies, decreasing fall incidences. Another promising area is in predicting adverse drug reactions in older adults on polypharmacy, a situation often overlooked. Anticipating these reactions might influence modfications and optimisation in therapy, reducing potential harm.

In light of the potential applications of machine learning to improve geriatric care, this review aims to bridge the gap in the literature by offering a comprehensive examination of machine learning's role in predicting clinical outcomes for older adults, particularly those ≥ 65 years, considering their increased healthcare utilisation and the scarcity of research tailored to this cohort.

## Methods

This systematic review was conducted after the study protocol was registered with PROSPERO (CRD42021295956).

### Search strategy

For this systematic review and meta-analysis, studies describing the use of machine learning models in predicting clinical outcomes in older adults (≥ 65 years) were assessed for the performance of machine learning models in predicting clinical outcomes in older adults (≥ 65 years old). The literature databases included in the study were PubMed, Embase, Web of Science core collection, Web of Science BIOSIS citation index, Scopus and ProQuest, using keywords in titles, abstracts, and index terms. This systematic review and meta-analysis used all relevant literature published in English up to the 28th of February, 2023. All studies were uploaded to EndNote Version 9 for duplicate removal. After removing the duplicates, the remaining studies were uploaded to Covidence software for abstract screening, full-text review, data extraction and quality assessment. The complete search strategy can be viewed in Supplementary Information Table 1.

### Inclusion and exclusion criteria

Predefined criteria were established for the inclusion and exclusion of studies. Regarding the participants, only older adults (years of age ≥ 65) were included. Studies concerning adult-only populations, animal studies, and pre-term populations were excluded. Regarding the intervention, studies which applied machine learning-based clinical prediction algorithms in older adults were included. Any synonyms for machine learning, such as “deep learning” or “statistical learning”, were included. The application of machine learning to non-clinical settings, the application of machine learning solely to images and/or signals, and studies that did not use machine learning were excluded. Regarding comparators, studies concerning machine learning vs other machine learning methods, machine learning vs traditional statistical, clinical prediction tools, and machine learning vs the unaided clinician were included. Studies utilising only traditional statistical prediction tools or unassisted clinician performance alone were excluded. Regarding the type of study, cohort studies (retrospective and prospective), cross-sectional studies and grey literature about the implementation of machine learning in clinical prediction tools in the geriatric setting were included. Narrative reviews, letters, abstracts only, corrigendum, no full-text available studies, and practice guidelines were excluded.

### Study selection process

The entire study review process, except duplicate removal, was conducted in Covidence software. RO conducted title and abstract screening and the full-text review. During the full-text review, a 10% sample of studies was blindly assessed by RO, SR, and PN to generate an agreement score, Cohen’s kappa score. Agreement reached 99.29%, and Cohen’s kappa score, 0.89, signifying almost perfect agreement.

### Data extraction and model performance

The data was extracted via Covidence into an Excel spreadsheet by RO and verified by SR and PN. The following items were extracted: Title, authors, in-text reference, journal, country, study design, participants/datasets used, participants/datasets sample size, primary outcome, machine learning approach, model assessment metric, statistical methods, study limitations, participant missing data, reasons for missing data. Corresponding authors of five original articles were contacted due to missing data, and one clarifying response was received. Papers for which a response was not received [17–20] were excluded from the meta-analysis. Studies which did not qualify for the meta-analysis underwent a narrative description.

The performance of a model is most commonly assessed via several performance metrics such as the Area Under the Receiver Operator Characteristic Curve (AUC-ROC), c-statistic, accuracy, F1 score, sensitivity, specificity, Positive Predictive Value (PPV), and Negative Predictive Value (NPV) [21]. It should be noted that assessing any given model using multiple metrics is generally recommended, ensuring a comprehensive analysis, as each metric has its limitations.

### PROBAST quality and risk of bias assessment

The Prediction model study Risk Of Bias Assessment Tool (PROBAST) was used to assess the quality of the studies included in this systematic literature review. PROBAST assesses both the risk of bias and concerns of applicability of a study aiming to develop, validate or update a multivariable diagnostic or prediction model. It is a tool suitable for the assessment of studies that utilise machine learning to predict clinical outcomes. PROBAST utilises four steps; step 1, in which the assessor specifies their systematic review question, step 2, in which the assessor classified the type of prediction model evaluation, step 3, in which the assessor judges the risk of bias and applicability within the model and step 4 in which the assessor passes their overall judgement on the model. RO and SR carried out the PROBAST risk of bias assessment, with PN as a tie-break.

### Statistical analyses

We conducted a random-effects meta-analysis using JASP software [22] based on the DerSimonian Laird model. Forest and funnel plots were also generated via JASP. In instances where the 95%CI for AUC-ROC results were absent, we estimated them. When only sensitivity and specificity were provided, we approximated an AUC-ROC value for a single point. Depending on the ROC curve type, the AUC_max_ can be approximated as the mean of the maximum and minimum areas.The formulas for approximations of 95%CI, single point AUC-ROC, AUC_max_ and the deviations from the PROSPERO protocol can be seen in Supplementary Information Appendix 1: Methods (cont).

## Results

### Study identification

**Fig. 1:**
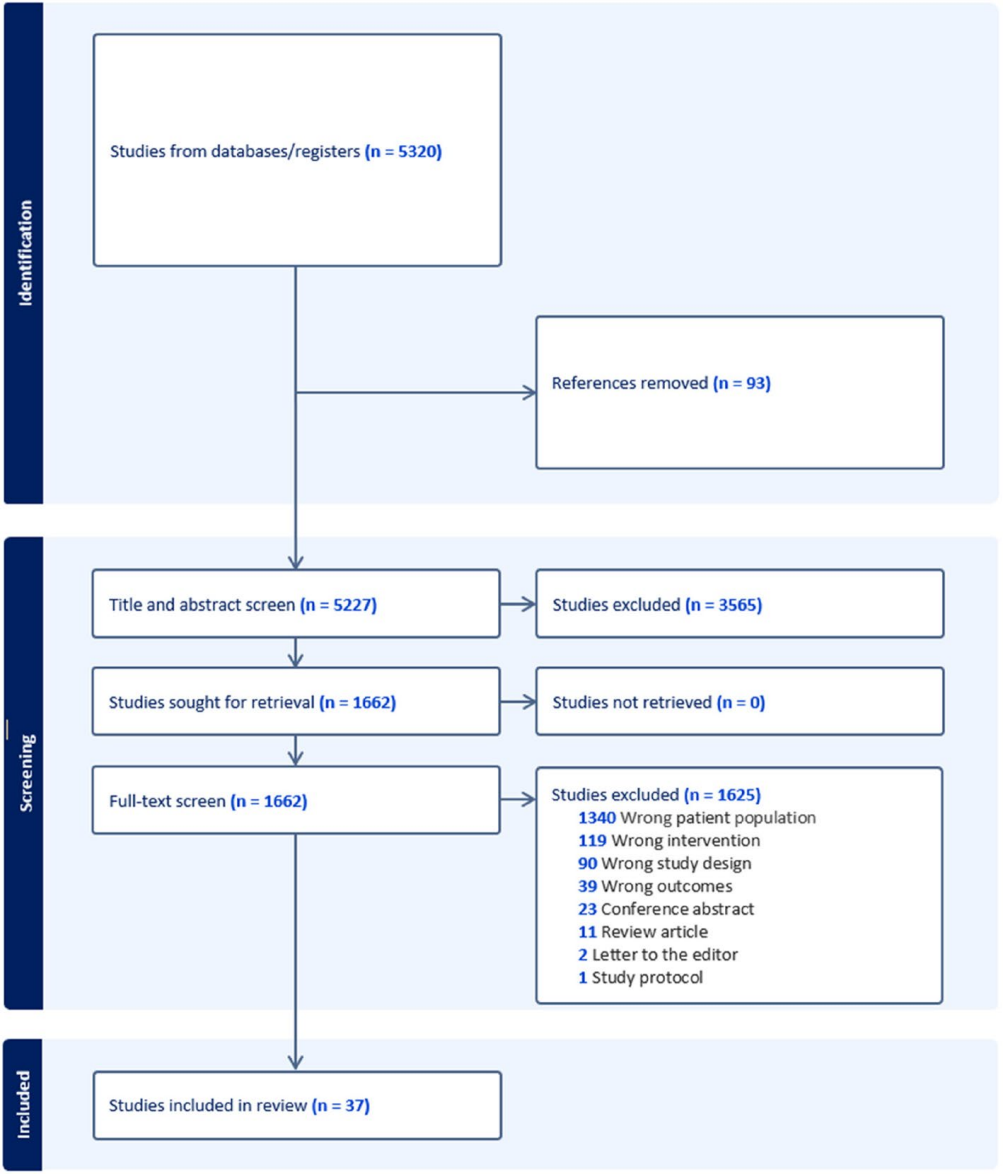
Study selection flowchart (PRISMA)

The search identified 11,185 studies. After removing duplicates, 5,227 studies underwent title and abstract screening. From the title and abstract screen, 1,662 studies progressed to full-text review, and 3,565 were considered irrelevant. From the full-text review, 37 studies progressed to data extraction, and 1,625 were excluded. The reasons for exclusion were the wrong patient population (*n* = 1,340), wrong intervention (*n* = 119), wrong study design (*n* = 90), wrong outcomes (*n* = 39), conference abstract (*n* = 23), review article (*n* = 11), letter to the editor (*n* = 2), study protocol (*n* = 1). Figure 1 shows the study selection flowchart.

### Study characteristics

The characteristics of the studies included in this review are summarised in Table 1. Most studies (*n* = *16*) originated from the USA, with the remaining studies originating from various EU countries as well as Taiwan, Japan, China and Iceland. All studies were published between 1994–2023, with the majority of studies published in the last five years (*n* = 29). The data was mainly acquired from EHRs (*n* = *20*). The majority of studies were of a retrospective design. The most common study design was retrospective cohort studies (*n* = *20*), and the least common study design was a descriptive study, longitudinal prospective study and retrospective case–control study (*n* = *1* each). There was a large variation in sample sizes, ranging from 15 to 1,214,489 patients. All studies concerned exclusively patients aged ≥ 65 years.

**Table 1:** Table of study characteristics

Authors; Year; Country	Study Design	Data Source	Sample Size	Age Group	Primary Outcome	ML Approach	ML Assessment Metric
Abdul Ghffar et al.; 2020; USA [20]	Case Control Study	Transcatheter Valve Therapy registry	354	Cohort 1 range 74–86 years, cohort 2 range 75–84 years	In-hospital cardiovascular mortality, In-hospital all-cause mortality, 30-day cardiovascular mortality, 30-day all-cause mortality	Decision trees, ensembles, logistic regressions, and deep nets fused into a single OptiML model	Specificity, sensitivity, false-negative rate, and AUC-ROC
Belmin et al., 2022; France [11]	Non-Randomised Controlled Trial	EHR	206	> 75 years, mean age 85 years	Risk of an emergency department visit within 14-days	RF	Gini index, AUC-ROC, local AUC-ROC
Bories et al., 2022; France [23]	Cohort Study	EHR	7,462	≥ 65 years	Hospitalisation for bleeding events	RF, SVM, XGBoost	AUC-ROC, accuracy, specificity, sensitivity
Bowen et al.; 2021; USA [12]	Case Control Study	EHR	15	Mean age 75.5 years	Pain perception in the last 7 days	Multilevel mixed modeling	AUC-ROC
Chen et al.; 2020; China [24]	Case Control Study	SEER database	964	Median age 67 years	Survival in patients with early stage uterine papillary serous carcinoma	Proportional subdistribution hazards regression	The c-index
Chung et al.; 2020; Taiwan [25]	Retrospective Cohort Study	EHR	31	Cohort 1 mean age 85.2 years, Cohort 2 mean age 83.9 years	mRS at 3-months, favourable/unfavourable clinical outcome at 3 months following AIS	Two ANN models	AUC-ROC, correlation efficiency, MSE
Considine et al.; 2019; Australia [26]	Prospective Case Control Study	EHR	1,717	Age range 70–87 years	Emergency interhospital transfer from subacute to acute care	Multivariable logistic regression	AUC-ROC, RMSE, mean absolute errors, AIC, BIC, Hosmer–Lemeshow statistics
Das et al.; 2003; USA [17]	Case Control Study	EHR	190	Mean age 75.5 years	Intervention for control of haemorrhage, recurrent bleeding, and death	ANN, multiple-logistic regression (BLEED)	Accuracy, sensitivity, specificity, PPV, NPV, likelihood ratios for positive and negative tests, discriminant power. McNemar's test, AUC-ROC
Diaz-Ramirez et al.; 2021; USA [27]	Case Control Study	Case-study data from HRS	5,531	≥ 70 years	Multiple outcomes, including nursing home admission as well as mortality	baBIC	Monte Carlo standard error, c-statistic
Duarte et al.; 2015; USA [28]	Case Control Study	EHR	467	≥ 65 years	6-month mortality risk	PROMPT	AUC-ROC, Hosmer–Lemeshow statistic, sensitivity, specificity, PPV, NPV, positive and negative likelihood ratios
Falsetti et al.; 2021; Italy [29]	Retrospective Cohort Study	EHR	1,326	The green group mean age 83 years, blue group mean age 79 years	Therapeutic failure, stroke/TIA and major bleeding	XGBoost	AUC-ROC
Ford et al.; 2021; UK [30]	Case Control Study	CPRD GOLD database	46,713	≥ 65 years	Dementia diagnosis code	Logistic regression, naive Bayes, RF	AUC-ROC, PPV
Fransvea et al., 2022; Italy [31]	Retrospective Cohort Study	FRAILESEL study	2,570	≥ 65 years	30-day mortality	Elastic-Net, SVM, KNN, Decision Tree, Multilayer Perceptron	Sensitivity, specificity
Friz et al., 2022; Italy [32]	Retrospective Cohort Study	EHR	3,079	≥ 65 years	All-cause 30-day readmission	Adaptive Boosting, Gradient Boosting, XGBoost, RF	PPV, NPV, AUC-ROC
Gomes et al.; 2021; Germany [33]	Case Control Study	EHR	451	Mean age 82.7 years	All-cause intrahospital mortality	ANN, SVM, RF	AUC-ROC
Han et al.; 2012; USA [34]	Descriptive Study	Data from four MHOS cohorts	21,870	≥ 65 years	6-month mortality	PROMPT	k-fold cross-validation, AUC-ROC, specificity, PPV, NPV, positive and negative likelihood ratios
Ko et al.; 2014; USA [35]	Retrospective Cohort Study	CORI data warehouse	14,844	≥ 65 years	Colonoscopy indication	Decision trees, linear discriminant analysis	Accuracy, sensitivity, specificity
Li Kuan Ong et al., 2023; Sigapore, UK, Australia [36]	Retrospective Cohort Study	EHR	150	Mean age at diagnosis: 71 years	Grade 1 and 2 genitourinary toxicity measured at 2 years post-radiotherapy follow-up	3 multivariate analysis models	AUC-ROC, Sensitivity, 1-specificity
Maurer et al., 2023; USA [37]	Retrospective Cohort Study	ACS-NSQIP database	29,366	≥ 65 years	30-day mortality	POTTER	Concordance statistics, AUC-ROC
Morris et al.; 2020; USA [38]	Case Control Study	The National Trauma Databank	1,214,489	≥ 65 years	In-hospital mortality	qEMAT, fEMAT	Calibration and AUC-ROC
Ocagli et al.; 2021; Italy [39]	Retrospective Cohort Study	EHR	78	≥ 65 years	4AT delirium score	RF	RMSE
Parenica et al.; 2012; Czech Republic [40]	Cohort Study	EHR	45	Mean age 82 years (75–89)	Adverse clinical outcomes after TAVI and SAVR	EuroSCORE	AUC-ROC
Pilotto et al.; 2010; Italy [41]	Retrospective Cohort Study	EHR	376	≥ 65 years	1-month mortality	MPI, NYHA, EFFECT, ADHERE	AUC-ROC
Pompei et al.; 1994; USA [42]	Retrospective Cohort Study	EHR	432	≥ 70 years	Delirium	Logistic regression	AUC-ROC
Ren et al., 2022; China [43]	Retrospective Cohort Study	Data from a large-scale prospective observational cohort study	2,526	≥ 65 years	Occurrence of various complications within 30 days of admission	RF	Accuracy, sensitivity, and specificity
Rossi et al.; 2021; Italy [13]	Retrospective Cohort Study	Patients from 2 population-based studies	1,794	≥ 80 years	Probabilities of developing myeloid neoplasms	Multivariable cox analysis	C-index, PPV, NPV, AUC-ROC
Sancarlo et al.; 2012; Italy [44]	Prospective Cohort Study	EHR	654	Mean age 79.3 years, range 66—99 years	1/6/12-month all-cause mortality	MPI	AUC-ROC
Sax et al.; 2021; USA [45]	Retrospective Cohort Study	EHR	26,189	Mean age 74 years	Serious adverse events within 30 days of emergency department arrival	Logistic regression, LASSO, decision tree, RF, and XGBoost	AUC-ROC, sensitivity, specificity, negative and positive likelihood ratios, PPV, NPV, F1 values
Shardell et al.; 2021; USA [46]	Retrospective Cohort Study	Patients from 5 large prospective, population-based studies	16,388	≥ 65 years	Sex-specific serum 25-hydroxyvitamin D thresholds that best discriminated incident slow gait	Weighted decision trees	Sensitivity
Suzuki et al.; 2020; Japan [18]	Retrospective Cohort Study	EHR	504	≥ 75 years	180-day all-cause mortality	Multiple logistic regression	AUC-ROC
Thongprayoon et al., 2023; USA, Thailand [47]	Retrospective Cohort Study	OPTN/UNOS	419	≥ 80 years	Distinct clusters of patients and their post-transplant outcomes including death-censored graft failure, mortality and acute allograft rejection	Unsupervised consensus clustering	Mean cluster consensus scores, area beneath the cumulative distribution function curves
Velagapudi et al.; 2021; USA [48]	Cross Sectional Study	EHR	220	≥ 65 years	Thrombolysis in cerebral infarction on first pass	Logistic regression, RF, SVM, Naive Bayes, and XGBoost	AUC-ROC, accuracy scores, Matthews correlation coefficient, Brier score, sensitivity, specificity
Venerito et al., 2022; Italy [49]	Retrospective Cohort Study	Primary data collected at medical centres in Bari, Firenze, Potenza and Sien	107	Mean age 74.1 years	3-month giant cell arthritis flare	Logistic Regression, simple Decision Tree, RF	AUC-ROC, Accuracy, Recall, Precision
Venturini et al.; 2021; Italy [19]	Retrospective Cohort Study	EHR	176	Median age 75.0 years	Various clinical outcomes including mortality, intensive care unit admission and intensive care unit discharge	Conditional RF, RF, Ordinal forest, Partitional tree, Conditional inference tree	Accuracy, sensitivity, specificity
Verdu-Rotellar et al., 2022; Spain, France, Slovenia, Bulgaria, Croatia, Hungary, Irealnd, Germany, Itlay, Sweden [50]	Retrospective Cohort Study	HEFESTOS	811	Mean age 82.2 years	30-day mortality, 30-day hospitalisation	Multivariable logistic regression	AUC-ROC
Zarkowsky et al.; 2021; USA [51]	Retrospective Cohort Study	The 'Vascular Quality Initiative Endovascular Aneurysm Repair' and 'National Surgical Quality Improvement' datasets	25,376	≥ 66 years	Short-stay discharge appropriateness, survival at 30 days	Multivariable statistical analyses, multilayer perceptron	AUC-ROC, Harrells c-statistic
Zhou et al.; 2021; USA [52]	Retrospective Cohort Study	The Framingham Heart Study data	149	Mean age 75.02 years	Heart failure with preserved ejection fraction risk	GA-KPLS, LASSO, RF, ridge regression, SVM, and logistic regression	AUC-ROC, sensitivity, specificity, accuracy, Youden index, G-means, and Matthews correlation coefficient, one-way ANOVA, Dunnetts multiple-comparison test

*Abbreviations*: *C-statistic* Concordance statistic, *NPV* Negative Predictive Value, *ANOVA* Analysis of Variance, *mRS* Modified Rankin Scale, *CORI* Clinical Outcomes Research Initiative, *MHOS* Medicare Health Outcomes Survey, *GA-KPLS* Kernel partial least squares with the genetic algorithm, *SEER* Surveillance, Epidemiology, and End Results, *HRS* Health and Retirement Study, *ADHERE* Acute Decompensated Heart Failure National Registry, *AIS* Acute Ischemic Stroke, *CPRD* Clinical Practice Research Datalink, *EFFECT* Enhanced Feedback for Effective Cardiac Treatment, *NYHA* New York Heart Association, *TIA* Transient Ischaemic Attack

### Outcomes

The primary outcome varied across studies, with most (*n* = *14*) predicting a mortality outcome. Other commonly reported outcomes included clinical care admission/readmission (*n* = *4*), delirium (*n* = *2*) and survival (*n* = *2*). Due to a limited number of studies, a meta-analysis was only performed for the mortality outcome, split into mortality within 6 months and mortality within a period longer than 6 months.

### Quality assessment

All studies included in this systematic review were evaluated for risk of bias and concerns regarding applicability using the PROBAST tool. Overall, the risk of bias in the majority of studies was considered low. The number of participants was a concern, with some studies utilising the data of fewer than 100 participants (*n* = *4*). Models built on such a small number of participants are unsuitable for external validation. Additionally, the current guidelines for reporting machine learning studies are unclear. Many studies are not accurately reporting key information about the model development or internal validation procedures. A summary table of the results from the PROBAST assessment can be seen in Supplementary Information Table 2.

### ML models

A wide variety of machine learning approaches were used across the studies. The most common machine learning approaches utilised included Random Forest (RF) (*n* = *12*), logistic regression (*n* = *11*), decision trees (*n* = *7*), XGBoost (*n* = *5*) and Artificial Neural Network (ANN) (*n* = *3*).

### ML model performance and evaluation

All studies utilised a classical approach for appraising the performance of the model. Most commonly, AUC-ROC was utilised in 28 studies. Other commonly used methods for appraising performance included sensitivity (*n* = *13*), specificity (*n* = *13*), accuracy (*n* = *8*), Root Mean Square Error (RMSE) (*n* = *2*), Mean Square Error (MSE) (*n* = *1*), and F1 values (*n* = *1*).

### Meta-analysis

**Fig. 2:**
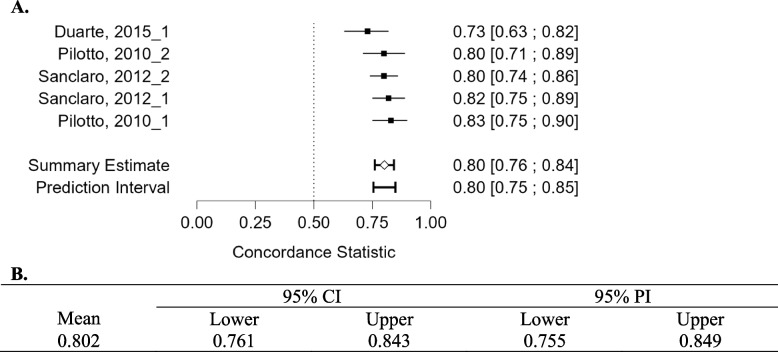
**A**. Forest plot comparing the c-statistic (AUC-ROC) from three studies included in the meta-analysis concerning models predicting mortality within 6 months or less. **B**. Concordance statistic meta-analysis summary

**Fig. 3:**
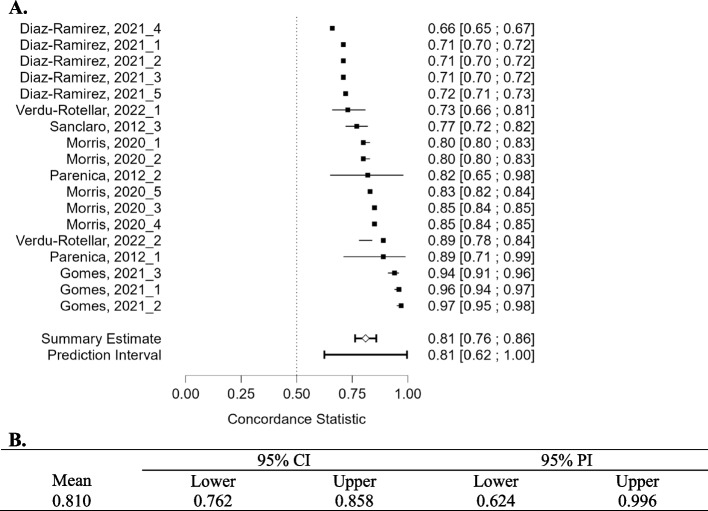
**A**. Forest plot comparing the c-statistic (AUC-ROC) from six studies included in the meta-analysis concerning models predicting mortality over 6 months or more. **B** Concordance statistic meta-analysis summary

Eight studies were included in the meta-analysis, which was split into two groups. Studies were grouped by the outcome (mortality) and compared based on the AUC-ROC. The summative results can be seen in Figs. 2A, B and 3A, B. Typically AUC-ROC values between 1—0.9 are considered ‘excellent’, 0.9—0.8 ‘good’, 0.8–0.7 ‘fair’, 0.7–0.6 ‘poor’, and 0.6–0.5 ‘failed’. The highest AUC-ROC were recorded by *Gomes *et al*., 2021*, where ANN, Support Vector Machine (SVM) and RF models were applied to predict all-cause intrahospital mortality after Transcatheter Aortic Valve Implantation (TAVI). The most effective model was RF with an AUC-ROC of 0.97 [95%CI: 0.95–0.98], followed by ANN (AUC-ROC of 0.96 [95%CI: 0.94–0.97]) and SVM (AUC-ROC of 0.94 [95%CI: 0.91–0.96]) [33]. Interestingly, the summary estimates for both meta-analyses were comparable, 0.80 [95%CI 0.76–0.84] for 6 months or less and 0.81 [95% CI 0.76–0.86] for 6 months or more. The models presented by *Diaz-Ramirez *et al*., 2021* showed the lowest AUC-ROC readings from the meta-analysis, ranging from an AUC-ROC of 0.66 for predicting mortality using the intersection method to 0.72 for predicting mortality using the full method model [27]. Several studies, such as *Duarte *et al*., 2015* [28], *Sancarlo *et al*., 2012* [44] and *Pilotto *et al*., 2010* [41] showed a large variety of AUC-ROC results. The complete key for Fig. 2 can be seen in Supplementary Information Appendix 2.

### Predictive model methods (mortality—within 6 months)

*Pilotto *et al*., 2010* applied Multidimensional Prognostic Index (MPI) to predict 1-month mortality in 376 patients with a diagnosis of heart failure split into cohorts by sex. Traditional regression model scores were also calculated. However, MPI performed best, reaching an AUC-ROC of 0.80 in women and 0.83 in men [41]. In a study by *Sancarlo *et al*., 2012* MPI was used to predict 1-month and 6-month all-cause mortality. MPI showed significantly high discriminatory power with AUC-ROC of 0.819, 0.799, respectively [44]. *Duarte *et al*., 2015* describe the Patient-Reported Outcome Mortality Prediction Tool (PROMPT) in predicting 6-month mortality. Patients were split into derivation/validation cohorts. PROMPT achieved similar AUC-ROC readings in both cohorts, 0.75 and 0.73, respectively. The authors concluded that PROMPT demonstrates good discrimination but poor calibration in independent heterogeneous datasets [28].

### Predictive model methods (mortality—over 6 months)

In the meta-analysis predicting mortality over 6 months, the top three models were from *Gomes *et al*., 2021*: RF (AUC-ROC 0.97), SVM (AUC-ROC 0.94), and ANN (AUC-ROC 0.96) to predict intrahospital mortality post-TAVI with 451 patients using 83 features. When restricted to 50 baseline features, performance dropped: AUC-ROC 0.81, 0.83, and 0.72, respectively. Traditional logistic risk scores lagged, scoring AUC-ROC 0.64 and 0.65 [33]. This suggests more input features improve AUC-ROC, but the study's limited patient sample limits its generalisability. *Verdu-Rotellar *et al*., 2022*, with a sample size of 811, applied multivariable logistic regression to predict risk in heart failure patients, attaining AUC-ROC scores of 0.73 and 0.89 in validation and derivation cohorts, respectively [50].

*Parenica *et al*., 2012* used the EuroScore on small TAVI and TAVI + SAVR cohorts of 29 and 42 patients respectively, achieving AUC-ROC 0.885 and 0.817 [40]. *Morris *et al*., 2020* designed a two-tiered mortality prediction system: qEMAT (on presentation) and fEMAT (post-radiologic evaluation). Both scored AUC-ROC of 0.80 and 0.85 in respective cohorts. In an external dataset, qEMAT recorded AUC-ROC 0.82 [38]. *Sancarlo *et al*., 2012*'s MPI model for 12-month mortality had AUC-ROC 0.770, a slight decrease from their short-term mortality predictions [44]. *Diaz-Ramirez *et al*., 2021* evaluated various Bayesian models, with the 'Full Method' showing the best performance at AUC-ROC 0.72. The 'Intersection method' scored the lowest, at AUC-ROC 0.66 [27].

**Fig. 4:**
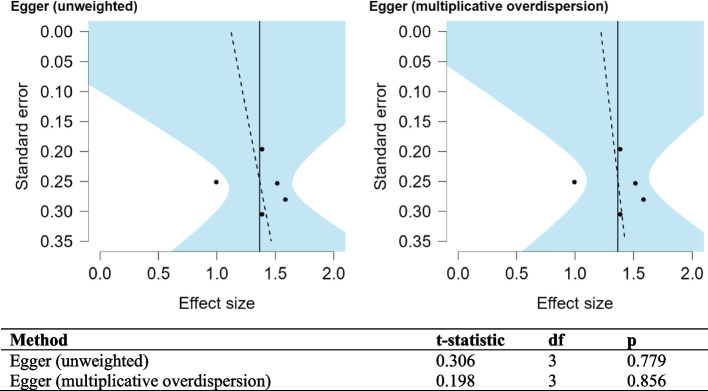
Funnel plot asymmetry test and asymmetry plots concerning models predicting mortality within 6 months or less

**Fig. 5:**
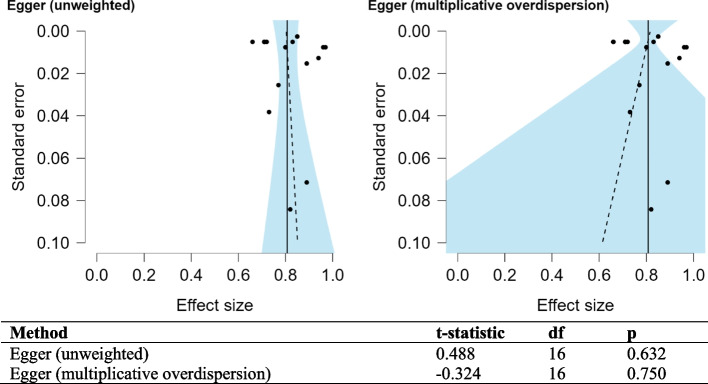
Funnel plot asymmetry test and asymmetry plots concerning models predicting mortality over 6 months or more

Funnel plots for the studies included in the meta-analysis show the variability of individual studies (standard error) versus the mean effect size, as seen in Figs. 4 and 5. For both meta-analyses, there is good symmetry in the unweighted and multiplicative overdispersion plots, with an equal dispersion of studies below and above the mean. Moreover, some studies fall outside the funnel in the unweighted and multiplicative overdispersion plots, indicating publication bias. It must be noted that Egger’s test [53] is less precise in detecting bias, given the small number of studies, particularly in the meta-analyses concerning mortality within 6-months. Prediction intervals (95% PI) can be seen in Figs. 2B and 3B. 95% PI values are useful because there is uncertainty associated with a single point value, to sum up the performance of a machine learning model. A range is much more useful when anticipating how a future prediction might perform. A 95% PI of over 0.6 on the lower bound and 0.8 on the upper bound in both meta-analyses suggest that the performance of the model in a validation study is expected to be good.

### Descriptive analysis of studies not included in the meta-analysis

Studies excluded from the meta-analysis also provide valuable information about the use of machine learning in predicting clinical outcomes. These studies were included in the meta-analysis because they aimed to predicts outcomes other than mortality but were too few in quantity to incorporate into a meta-analysis and directly compare the effect sizes. For a tabular representation of study characteristics, please see Table 1. Studies by *Abdul-Ghaffar *et al*., 2020* [20], *Das *et al*., 2003* [17], *Fransvea *et al*., 2022* [31], *Maurer *et al*., 2023* [37], *Suzuki *et al*., 2020* [18], *Venturini *et al*., 2021* [19]*, Ghotra *et al*., 2020* [54]* and Han *et al*., 2012* [34] concerned mortality outcomes, but missing information prevented us from utilising these studies in the meta-analysis. The studies in this section have been grouped by primary outcome.

### Studies predicting mortality outcomes

*Abdul Ghaffar *et al*., 2020* combined decision trees, ensembles, logistic regression and deep nets into a single OptiML model to predict in-hospital mortality outcomes in post-TAVI patients. For 30-day cardiovascular mortality, the phenogroup data combined with the Society of Thoracic Surgeons (STS) score improved discriminatory power compared to the STS score alone (AUC-ROC 0.96 vs 0.80). In conclusion, the group showed that applying OptiML could identify patients at higher risk of various types of mortality post Tpost-TAVI [20]. *Das *et al*., 2003* compared an ANN model with a BLEED model for the prediction of death, recurrent bleeding, and therapeutic interventions for the control of haemorrhage. The ANN model significantly outperformed the BLEED model in terms of accuracy for predicting death (97% vs 70%), recurrent bleeding (93% vs 73%) and need for interevention (93% vs 70%) during the external validation. The ANN model showed good discriminatory power and was concluded to have benefits for risk stratification in these populations [17]. *Fransvea *et al*., 2022* predicted 30-day mortality using five machine learning models; elastic net, SVM, KNN, decision tree, multilayer perceptron. The best performance was observed using the multilayer perceptron model, attaining an accuracy of 94.9% and outperforming traditionally used approaches [31]. *Han *et al*., 2012* developed a PROMPT model for the prediction of 6-month mortality in community-dwelling older adults. At 10%-70% cutpoints, PROMPT achieved a sensitivity of 0.80%-83.4%. The models’ discriminatory power was further supported by the fact that over half of patients with estimated 6-month mortality > 30% died within a 12-month follow-up period [34]. *Maurer *et al*., 2023* performed an external validation of a Predictive OpTimal Trees in Emergency Surgery Risk (POTTER) model, predicting 30-day mortality. POTTER is available as a mobile telephone app and outperforms all traditional risk prediction approaches. The model achieved a c-statistic of 0.80, with its worst performance in the ≥ 80 years cohort (c-statistic = 0.71) and its best in the 65–74 years cohort (c-statistic = 0.84). The group concluded that POTTER could be useful in emergency surgery departments [37]. *Suzuki *et al*., 2020* developed a multiple logistic regression model to predict 180-day all-cause mortality. The model showed good discriminatory power with a c-statistic of 0.820. The group remarked on the models’ potential application in deciding the direction of therapy, palliative care or hospital referrals [18]. *Thongprayoon *et al*., 2023* utilised a clustering approach (unsupervised consensus clustering) to allocate patients and their post-transplant outcomes, such as mortality, graft failure and acute allgraft rejection. Graphical mean cluster consensus scores suggest good model performance [47]. *Venturini *et al*., 2021* developed five tree models designed for the prediction of various clinical outcomes, including mortality, intensive care unit admission and intensive care unit discharge. The accuracy scores recorded were as follows; conditional RF (0.70), RF (0.79), ordinal forest (0.77), partitional tree (0.65), and conditional inference tree (0.79). While these results show good discriminatory power, external validation is needed before implementation into clinical care [19].

### Studies predicting hospitalisation, admission, and transfer outcomes

*Belmin *et al*., 2022* utilised a previously developed machine learning algorithm to predict emergency department admissions using questionnaire data collected during home visits, achieving a sensitivity of 83%. The system was successfully implemented, and the group concluded that it could have a use in lowering the number of emergency department visits within the study cohort [11]. *Bories *et al*., 2022* built three machine learning models to predict hospitalisation for bleeding effects. The RF, XGBoost, and Support Vector Machine achieved predictive accuracy of 0.64, 0.68 and 0.64, respectively, in a sample of 7,462 participants [23]. *Considine *et al*., 2019* utilised multivariable logistic regression to predict emergency interhospital transfer from subacute to acute care in a cohort of 1717 participants. The model achieved a median AUC of 0.77. The group concluded that while the results from the model are encouraging, further development and testing is necessary before implementing for external validation [26]. *Friz *et al*., 2022* developed four machine learning models with good discriminatory power to predict 30-day readmission; adaptive boosting (AUC = 0.803), gradient boosting (AUC-ROC = 0.782), XGBoost (AUC-ROC = 0.776), RF (AUC-ROC = 0.786). All four models outperformed the traditionally used LACE index, which achieved an AUC of 0.504; however, they require external validation before introducing into a clinical setting [32]. *Ren *et al*., 2022* developed an RF model for predicting various hospital complications within 30 days of admission and investigating feature importance from three traditionally used risk scores [43]. *Sax *et al*., 2021* developed five machine learning models to predict serious adverse events within 30 days of emergency department admission. Logistic regression, Least Absolute Shrinkage and Selection Operator (LASSO), decision tree, RF, and XGBoost models achieved c-statistic of 0.80, 0.80, 0.65, 0.83, 0.85, respectively. The group concluded that using machine learning is an improvement on using traditional approaches [45]. *Zarkowsky *et al*., 2021* developed a multivariable statistical model and a multilayer perceptron to predict the appropriateness of short-stay discharge and survival at 30 days. Bootstrap model validation with 1000 repetitions generated an AUC-ROC of 0.71. The study found that selecting patients for short-stay discharge is possible without risk to the patient [51].

### Studies predicting cardiovascular outcomes

*Chung *et al*., 2020* developed two ANN models to predict i) favourable/poor clinical outcome ii) modified Rankin Scale at 3 months in a sample of participants with acute ischemic stroke. Both models performed well, predicting clinical outcomes with an AUC-ROC of 0.974 and predicting modified Rankin Scale at 3 months with a low error, MSE = 0.24. In conclusion, the group showed the usefulness of these two ANN models and remarked that they could benefit clinicians, assisting with therapeutic decision-making. It should be noted that this analysis included only 31 participants [25]. *Falsetti *et al*., 2021* developed an XGBoost model for the prediction of 3 clinical outcomes, all three models showing good discriminatory power: stroke/transient ischaemic attack (AUC-ROC = 0.931), therapeutic failure (AUC-ROC = 0.974), and major bleeding (AUC-ROC = 0.930). The models outperformed traditional risk scores, and the group concluded that the study was a big step in the instrumentation of machine learning in a larger clinical setting [29]. *Velagapudi *et al*., 2021* developed and optimised five machine learning model classifiers to predict thrombolysis in cerebral infarction on first pass. Logistic regression, RF, SVM, Naïve Bayes and XGBoost performed to an AUC of 0.657, 0.659, 0.642, 0.599 and 0.599, respectively. The authors remark that given the improved predictive power, ease of integration with new data and generalisability, machine learning approaches are preferred to traditional approaches for studying clinical outcomes in stroke populations [48]. *Zhou *et al*., 2021* developed six machine learning models to predict heart failure by identifying participant subgroups at high risk of death. Kernel partial least squares with the genetic algorithm (GA-KPLS), RF, LASSO, ridge regression, logistic regression and SVM achieved AUC-ROC results of 0.995, 0.646, 0.774, 0.734, 0.591, and 0.929, respectively. The results achieved with the GA-KPLS and SVM models are particularly high compared to others, which could be explained by a small participant count of just 149. The authors concluded that while the performance of the models was encouraging, external validation in another dataset/in a live clinical setting is required [52].

### Studies predicting other clinical outcomes

*Bowen *et al*., 2021* used a multilevel mixed model to predict pain perception in the last 7 days. The model included 58 continuous features and showed that poor sleep is highly associated with pain perception in the last 7 days. It should be noted that a sample size of just 15 community-dwelling older adults was used in this study, which is a significant limiting factor when considering how generalisable the results are in the larger geriatric care setting [12]. *Chen *et al*., 2020* predicted survival in patients with early-stage uterine papillary serous carcinoma using a proportional subdistribution hazards regression predictive model. The model showed great risk stratification ability as the participants placed in the high-risk-of-death group had higher incidence of death (*p* < 0.001). The group concluded that the model showed promising results [24]. *Ford *et al*., 2021* developed three models (logistic regression, naïve Bayes, RF) to detect dementia in patients where the symptoms were identified in primary care, but a formal diagnosis was not made. All three models achieved similar AUC-ROC results in the range of 0.87–0.90 with coded variables and 0.90–0.94 when keywords were added. The study showed the benefit of using machine learning for the retrospective diagnosis of dementia, assuring that records are up to date and that good quality of care is provided to patients [30]. *Ko *et al*., 2014* aimed to develop two machine learning models, decision trees and linear discriminant analysis, to classify colonoscopy indications from EHR data. The overall accuracy for classifying colonoscopy indication was 71%, 73%, and 68% for decision trees, linear discriminant analysis model 1 and linear discriminant analysis model 2, respectively. The study concluded that while these results are promising, external validation is necessary before deploying in clinical care [35]. *Li Kuan Ong *et al*., 2023* developed three multivariate analysis models to predict the risk of late genitourinary toxicity in older adults with prostate cancer receiving radiotherapy. Models 1, 1a and 2 achieved AUC of 0.63, 0.64, and 0.81, respectively [36]. *Ocagli *et al*., 2021* developed an RF model to predict 4AT delirium scores. The RF model predicted 4AT scores with an RMSE of 3.29. The study showed that RF is a valid method for predicting 4AT scores and assessing the factors associated with delirium [39]. *Pompei *et al*., 1994* aimed to develop and validate a logistic regression model for predicting the risk of delirium by classifying participants into one of three ascending risk groups. The model achieved good discriminatory power with AUC-ROC of 0.74 [42]. *Rossi *et al*., 2021* defined a multivariable Cox analysis predictive model to stratify patients into three groups based on the risk of developing myeloid neoplasms. The model achieved c-index scores of 0.851 and 0.889 in the internal and external validation cohorts. These results, coupled with a reasonable sample size of 1,794 patients, suggest a good generalisability of the results [13]. *Shardell *et al*., 2021* predicted sex-specific serum 25-hydroxyvitamin D thresholds that best discriminated incident slow gait using weighted decision trees [46]. *Venerito *et al*., 2022* developed three machine learning approaches to predict a 3-month giant cell arthritis flare-up. RF outperformed logistic regression and decision trees, with accuracy scores of 71.4%, 70.4%, and 62.9%, respectively. AUC-ROC RF outperformed logistic regression and decision trees 0.76, 0.73, and 0.65, respectively. The authors concluded that their approach is highly reproducible and capable of being a benefit to a clinician caring for an older adult [49].

## Discussion

Our review identified 37 studies which utilised a machine learning approach to predict a clinical outcome in adults aged ≥ 65 years. The two-grouped meta-analysis consisted of a total of eight studies using different machine learning models to predict mortality within 6 months in the first group and mortality within a period over 6 months for the second group. Supported by an AUC-ROC summary estimate of 0.80 (95% CI: 0.76 – 0.84) for mortality within 6 months and 0.81 (95% CI: 0.76 – 0.86) for mortality over 6 months, we found that machine learning models display good discriminatory power in predicting mortality. While the future of utilising machine learning in geriatric care looks favourable, caution must be applied before proclaiming it the new gold standard. This systematic literature review and meta-analysis identified some key issues facing machine learning applications in geriatric settings.

First, many studies have very low numbers of participants, some as low as under 100 participants. Machine learning models benefit greatly from large datasets numbering thousands of observations, with a low percentage of missing data. Second, many studies are utilising datasets of participants with varying levels of heterogeneity. Studies which use highly heterogeneous populations cannot be generalised to the general population of older adults. Third, most studies do not validate their findings externally. External validation studies incorporating a large number of participants are necessary before machine learning can be applied in the wider geriatric care setting. Fourth, ideally, machine learning models could be applied to any Electronic Healthcare Record (EHR)/clinical database and make live predictions as new data is introduced. However, this will prove challenging since many EHR differ fundamentally in data structures. Therefore, EHRs and clinical databases must be standardised to utilise these approaches fully. Fifth, some machine learning algorithms require much larger amounts of high-quality data to make accurate predictions than traditional statistical approaches. Sixth, most machine learning algorithms need to be optimised, and the optimal model tuning would be user dependent, which may introduce additional challenges in assessing each model’s performance and risk of bias. Finally, we found significant inconsistencies in reporting machine learning study findings. Many studies report limited information on model development and performance metrics with no insights into 95% CI. We recommend that journals specialising in this niche take special care in encouraging authors to be detailed in reporting their model development approach and encourage them to supply a full view of model performance metrics.

In 2020, a systematic literature review was carried out by *Choudhury *et al*. 2020*, in which the authors identified 35 eligible studies concerning machine learning in geriatric care for chronic diseases. Similar to the findings in this review, the group concluded that while the applications of machine learning in geriatric settings look promising, more validation studies are needed, and machine learning needs to be standardised and tailored to geriatric settings carefully [14].

### Limitations and strengths

Our approach has several limitations. First, the search was limited to English, meaning relevant literature in other languages was not considered. Second, the review could have missed some studies due to unusual terminology. To mitigate this, significant time was spent identifying all synonyms of keywords and working with an experienced subject librarian when designing the search strategy. Third, due to limited literature in the field, only eight studies were included in the meta-analysis. Fourth, there is much heterogeneity between the patients from the studies included in the meta-analysis, and several patients had co-morbidities such as heart failure. Additionally, we could not calculate an I^2^ due to the studies failing to report the standard error. Fifth, we could only pool studies by one outcome (mortality) as there were inconsistent definitions and sparse data to pool studies for other clinical outcomes. Further to this point, further grouping of patients into cohorts with similar comorbidities is necessary but was not carried out as part of this meta-analysis, given the limited number of papers. Grouping patients into such cohorts would greatly improve the generalisability of our results. Sixth, inconsistent reporting of the model development process and inconsistent reporting of model performance meant that some studies appropriate for meta-analysis were not included. For instance, not all performance metrics can be converted to AUC-ROC, especially without specific data on the number of subjects in the derivation/validation cohorts. Finally, the predictions were performed on retrospective data, mostly from electronic health records; validating these models on prospectively collected data from other sources is critical for reproducibility. Our study’s strengths include following PRISMA guidelines, having our protocol approved and registered by PROSPERO, using Covidence software (increased rigour, accurate documentation), using a highly appropriate quality of study tool (PROBAST) and utilising multiple literature databases.

## Conclusion

Given the vast amounts of clinical data collected from patients, there is considerable potential in utilising machine learning in predicting clinical outcomes in older adults. This review showed that machine learning models exhibit high discriminatory power, often outperforming traditional statistical approaches. It should also be considered that as datasets become larger and machine learning models become more sophisticated, their performance will increase further. As this field of research develops, we believe it is crucial that specific attention is dedicated to a comprehensive model performance analysis for each model. More large-scale validation studies are needed to show that machine learning can predict clinical outcomes in older adults underrepresented in our findings, such as predicting risk factors for dementia, identifying risk factors for multimorbidity, and screening subgroups of older adults vulnerable to falls and fractures.

### Supplementary Information

**Additional file 1:** **Supplementary Information Appendix 1.** Methods (cont). **Supplementary Information Appendix 2.** A key for Figure. 2 detailing the characteristics of the studies included in the meta analysis. **Supplementary Information Table 1.** Full search strategy. **Supplementary Information Table 2.** PROBAST assessment. **Supplementary Information Appendix 3.** PRISMA checklist.

## Data Availability

The datasets used and/or analysed during the current study available from the corresponding author on reasonable request.

## Abbreviations

95% CI: 95% Confidence Interval
95% PI: 95% Prediction Interval
ACS-NSQIP: ACS National Surgical Quality Improvement Program
ADHERE: Acute Decompensated Heart Failure National Registry
AIS: Acute Ischemic Stroke
ANN: Artificial Neural Network
ANOVA: Analysis of Variance
AUC: Area Under the Curve
AUC-ROC: Area Under the Receiver Operator Characteristic (ROC) Curve
baBIC: Best Average Bayesian Information Criterion
CORI: Clinical Outcomes Research Initiative
CPRD: Clinical Practice Research Datalink
EFFECT: Enhanced Feedback for Effective Cardiac Treatment
EHR: Electronic Healthcare Record
fEMAT: Full Elderly Mortality After Trauma
GA-KPLS: Kernel partial least squares with the genetic algorithm
HRS: Health and Retirement Study
LASSO: Least Absolute Shrinkage and Selection Operator
MHOS: Medicare Health Outcomes Survey
MPI: Multidimensional Prognostic Index
mRS: Modified Rankin Scale
MSE: Mean Square Error
NPV: Negative Predictive Value
NYHA: New York Heart Association
OPTN: Organ Procurement and Transplantation Network
POTTER: Predictive OpTimal Trees in Emergency Surgery Risk
PPV: Positive Predictive Value
PROBAST: Prediction model study Risk Of Bias Assessment Tool
PROMPT: Patient-Reported Outcome Mortality Prediction Tool
qEMAT: Quick Elderly Mortality After Trauma
RF: Random Forest
RMSE: Root Mean Square Error
SAVR: Surgical Aortic Valve Replacement
SEER: Surveillance, Epidemiology, and End Results
STS: Society of Thoracic Surgeons
SVM: Support Vector Machine
TAVI: Transcatheter Aortic Valve Implantation
TIA: Transient Ischaemic Attack
UNOS: United Network for Organ Sharing
