# Supplementary material for: Application of machine learning approaches in predicting clinical outcomes in older adults – a systematic review and meta-analysis
Source: BMC Geriatr. 2023 Sep 14;23:561. doi: 10.1186/s12877-023-04246-w (PMC10503191; doi:10.1186/s12877-023-04246-w)
Supplement: Supplementary file 1 — Additional file 1: Supplementary Information Appendix 1. Methods (cont). Supplementary Information Appendix 2. A key for Figure. 2 detailing the characteristics of the studies included in the meta analysis. Supplementary Information Table 1. Full search strategy. Supplementary Information Table 2. PROBAST assessment. Supplementary Information Appendix 3. PRISMA checklist. [file 12877_2023_4246_MOESM1_ESM.docx]

**Supplementary Information Index**

| Supplementary Information Appendix 1. Methods (cont) |  | Page 2 |
| --- | --- | --- |
| Supplementary Information Appendix 2. A key for Figure  2 detailing the characteristics of the studies included in the meta analysis. |  | Page 3 |
| Supplementary Information Table 1. Full search strategy. |  | Page 4-5 |
| Supplementary Information Table 2. PROBAST assessment |  | Page 6-9 |
| Supplementary Information Appendix 3. PRISMA checklist. |  | Page 10 |
| References |  | Page 11 |

**Supplementary Information Appendix 1. Methods (cont)**

The random effects meta-analysis was conducted in JASP software(1). DerSimonian Laird model was used. Forest plots and funnel plots were generated using JASP. In the case of missing 95%CI for AUC-ROC results, the following formula was used to estimate the 95%CI:

$$CI=AUC \pm Z_{1-\frac{\alpha}{2}} \times se$$

$$se= \sqrt{\frac{q_{0}+\left( n_{1}-1 \right)q_{1}+\left( n_{2}-1 \right)q_{2}}{n_{1}n_{2}}}$$

$q_{0}=AUC\left( 1-AUC \right) q_{1}=\frac{AUC}{2-AUC}-{AUC}^{2} q_{2}= \frac{{2AUC}^{2}}{1+AUC}-{AUC}^{2}$

An AUC-ROC value for a single point was estimated for instances where only sensitivity and specificity were reported. The AUC min was calculated geometrically using the following formula:

$${AUC}_{min}=\frac{( sensitivity+specificity )}{2}$$

For AUC max, depending on the type of receiver operating characteristic (ROC) curve, the AUC can be approximated as the means of the maximum and minimum area. In the formula below, *F* denotes the false positive rate, and *H* denotes the true positive rate.

$${AUC}_{max}=\begin{matrix} 1-2F\left( 1-H \right), & if F\leq0.5<H \\ 1-\frac{F}{2H}, & if F \leq H <0.5 \\ 1-\frac{1-H}{2\left( 1-F \right)}, & if 0.5 <F \leq H \end{matrix}$$

**Deviations from the PROSPERO Protocol**

There were three deviations from our original protocol registered in PROSPERO. Firstly, ‘PsychINFO’ and ‘Cochrane Central Register of Controlled Trials’ databases were not utilised due to a lack of relevant papers. Secondly, we chose to assess bias and study the quality of studies using the PROBAST method which is more appropriate for systematic reviews aiming to assess the performance of machine learning models in predicting clinical outcomes. Finally, due to a lengthy review process we have extended the inclusion criteria to February 2023, ensuring a more up-to-date cover of the available literature.

**Supplementary Information Appendix 2.** *A key for Figure 2 detailing the characteristics of the studies included in the meta-analysis.*

**Mortality within 6-months**

Duarte, 2015_1 - predicting 6-month mortality using PROMPT.

Pilotto, 2010_1 - predicting 1-month mortality in men using MPI combined with linear regression.

Pilotto, 2010_2 - predicting 1-month mortality in women using MPI combined with linear regression; Sanclaro, 2012_1 - predicting 1-month mortality using MPI.

Sanclaro, 2012_2 - predicting 6-month mortality using MPI.

**Mortality within 6-months or more**

Sanclaro, 2012_3 - predicting 12-month mortality using MPI.

Gomes, 2021_1 - predicting all-cause mortality using ANN.

Gomes, 2021_2 - predicting all-cause mortality using RF.

Gomes, 2021_3 - predicting all-cause mortality using SVM.

Morris, 2020_1 - predicting all-cause hospital mortality in the derivation cohort using qEMAT.

Morris, 2020_2 - predicting all-cause hospital mortality in the validation cohort using qEMAT.

Morris, 2020_3 - predicting all-cause hospital mortality in the derivation cohort using fEMAT.

Morris, 2020_4 - predicting all-cause hospital mortality in the validation cohort using fEMAT.

Morris, 2020_5 - predicting all-cause hospital mortality in the Michigan Trauma Quality Improvement Program cohort using qEMAT.

Diaz-Ramirez, 2021_1 - predicting mortality using the individual outcome method.

Diaz-Ramirez, 2021_2 - predicting mortality using the union method.

Diaz-Ramirez, 2021_3 - predicting mortality using the baBIC method.

Diaz-Ramirez, 2021_4 - predicting mortality using the intersection method.

Diaz-Ramirez, 2021_5 - predicting mortality using the full method

Verdu-Rotellar, 2022_1 – predicting risk of death or hospitalization (compound outcome) in the validation cohort using multiple logistic regression

Verdu-Rotellar, 2022_2 – predicting risk of death or hospitalization (compound outcome) in the derivation cohort using multiple logistic regression

Parenica, 2012_1 – predicting 1-year mortality in the TAVI cohort using EuroScore

Parenica, 2012_2 – predicting 1-year mortality in the SAVR+TAVI cohort using EuroScore

**Supplementary Information Table 1A.** *Full search strategy.*

| **Database** | **Search #** | **Search query** | **Results** |  |
| --- | --- | --- | --- | --- |
| **Embase** | #1 | 'machine learning*':ti,ab,kw OR 'artificial intelligence*':ti,ab,kw OR 'deep learning*':ti,ab,kw OR 'statistical learning*':ti,ab,kw OR algorithm*:ti,ab,kw OR 'expert system*':ti,ab,kw OR 'neural network*':ti,ab,kw OR 'natural language processing*':ti,ab,kw OR 'predictive model*':ti,ab,kw | 533,598 |  |
|  | #2 | 'clinical outcome*':ti,ab,kw OR 'critical care outcome*':ti,ab,kw OR 'patient-reported outcome*':ti,ab,kw | 150,193 |  |
|  | #3 | 'older adult*':ti,ab,kw OR 'over 65*':ti,ab,kw OR 'elderly*':ti,ab,kw OR 'geriatric*':ti,ab,kw OR 'over 80*':ti,ab,kw OR ‘aged*’:ti,ab,kw OR 'aged'/exp OR 'very elderly'/exp | 4,107,851 |  |
|  | **#4** | **#1 AND #2 AND #3** | **602** |  |
| **PubMed** | #1 | (((((((((((((((((machine learning* [Title/Abstract]) OR (artificial intelligence*[Title/Abstract])) OR (deep learning*[Title/Abstract])) OR (statistical learning*[Title/Abstract])) OR (algorithm*[Title/Abstract])) OR (expert system*[Title/Abstract])) OR (neural network*[Title/Abstract])) OR (natural language processing*[Title/Abstract])) OR (predictive model*[Title/Abstract])) OR (machine learning*[Text Word])) OR (artificial intelligence*[Text Word])) OR (deep learning*[Text Word])) OR (statistical learning*[Text Word])) OR (algorithm*[Text Word])) OR (expert system*[Text Word])) OR (neural network*[Text Word])) OR (natural language processing*[Text Word])) OR (predictive model*[Text Word]) | 587, 901 |  |
|  | #2 | ((((((clinical outcome*[Title/Abstract]) OR (critical care outcome*[Title/Abstract])) OR (patient-reported outcome*[Title/Abstract])) OR (clinical outcome[Text Word])) OR (clinical outcome*[Text Word])) OR (critical care outcome*[Text Word])) OR (patient-reported outcome*[Text Word]) | 231,062 |  |
|  | #3 | (((((((((((((older adult*[Title/Abstract]) OR (over 65*[Title/Abstract])) OR (elderly*[Title/Abstract])) OR (geriatric*[Title/Abstract])) OR (over 80*[Title/Abstract])) OR (aged*[Title/Abstract])) OR (very elderly*[Title/Abstract])) OR (older adult*[Text Word])) OR (over 65*[Text Word])) OR (elderly*[Text Word])) OR (geriatric*[Text Word])) OR (over 80*[Text Word])) OR (aged*[Text Word])) OR (very elderly*[Text Word]) | 5,889,093 |  |
|  | **#4** | **#1 AND #2 AND #3** | **1,831** |  |
| **Web of Science Core collection** | #1 | TS = ("machine learning*" or "artificial intelligence*" or "deep learning*" or "statistical learning*" or "algorithm*" or "expert system*" or "neural network*" or "natural language processing*" or "predictive model*") | 2,937,326 |  |
|  | #2 | TS = ("clinical outcome*" or "critical care outcome* " or "patient-reported outcome*") | 256,594 |  |
|  | #3 | TS = ("older adult*" or "over 65*" or "elderly*" or "geriatric*" or "over 80*" or "aged*" or "very elderly*") | 1,127,319 |  |
|  | **#4** | **#1 AND #2 AND #3** | **208** |  |
| **Web of Science BIOSIS citation index** | #1 | TS = ("machine learning*" or "artificial intelligence*" or "deep learning*" or "statistical learning*" or "algorithm*" or "expert system*" or "neural network*" or "natural language processing*" or "predictive model*") | 298,050 |  |
|  | #2 | TS = ("clinical outcome*" or "critical care outcome* " or "patient-reported outcome*") | 126,219 |  |
|  | #3 | TS = ("older adult*" or "over 65*" or "elderly*" or "geriatric*" or "over 80*" or "aged*" or "very elderly*") | 1,569,198 |  |
|  | **#4** | **#1 AND #2 AND #3** | **693** |  |
| **Scopus** | #1 | ( TITLE-ABS-KEY ( "machine learning" ) OR TITLE-ABS-KEY ( "artificial intelligence*" ) OR TITLE-ABS-KEY ( "deep learning*" ) OR TITLE-ABS-KEY ( "statistical learning" ) OR TITLE-ABS-KEY ( "algorithm*" ) OR TITLE-ABS-KEY ( "expert system*" ) OR TITLE-ABS-KEY ( "neural network*" ) OR TITLE-ABS-KEY ( "natural language processing*" ) OR TITLE-ABS-KEY ( "predictive model*" ) ) | 4,365,173 |  |
|  |  |  |  |  |
|  | #2 | ( TITLE-ABS-KEY ( "clinical outcome*" ) OR TITLE-ABS-KEY ( "critical care outcome*" ) OR TITLE-ABS-KEY ( "patient reported outcome*" ) ) | 356,431 |  |
|  | #3 | ( TITLE-ABS-KEY ( "older adult*" ) OR TITLE-ABS-KEY ( "over 65*" ) OR TITLE-ABS-KEY ( "over 80*" ) OR TITLE-ABS-KEY ( "aged*" ) OR TITLE-ABS-KEY ( "elderly*" ) OR TITLE-ABS-KEY ( "very elderly*" ) OR TITLE-ABS-KEY ( "geriatric*" ) ) | 5,947,699 |  |
|  |  |  |  |  |
|  | **#4** | **#1 AND #2 AND #3** | **3,511** |  |
| **ProQuest** | #1 | ab(“machine learning*” OR “artificial intelligence*” OR “deep learning*” OR “statistical learning*” OR “algorithm*” or “expert system*” OR “neural network*” OR “natural language processing*” OR “predictive model*”) OR ti(“machine learning*” OR “artificial intelligence*” OR “deep learning*” OR “statistical learning*” OR “algorithm*” or “expert system*” OR “neural network*” OR “natural language processing*” OR “predictive model*”) | 748,457 |  |
|  | #2 | ab(“clinical outcome*” OR “critical care outcome*” OR “patient reported outcome*”) OR ti(“clinical outcome*” OR “critical care outcome*” OR “patient reported outcome*”) | 37,997 |  |
|  | #3 | ab(“older adult*” OR “over 65*” OR “elderly*” OR “geriatric*” OR “over 80*” OR “aged*” OR “very elderly*”) OR ti(“older adult*” OR “over 65*” OR “elderly*” OR “geriatric*” OR “over 80*” OR “aged*” OR “very elderly*”) | 415,856 |  |
|  | **#4** | **#1 AND #2 AND #3** | **37** |  |

**Supplementary Information Table 1B.** *Updated search strategy (up to February 2023).*

| **Database** | **Search #** | **Search query** | **Results** |  |
| --- | --- | --- | --- | --- |
| **Embase** | #1 | machine learning*':ti,ab,kw OR 'artificial intelligence*':ti,ab,kw OR 'deep learning*':ti,ab,kw OR 'statistical learning*':ti,ab,kw OR algorithm*:ti,ab,kw OR 'expert system*':ti,ab,kw OR 'neural network*':ti,ab,kw OR 'natural language processing*':ti,ab,kw OR 'predictive model*':ti,ab,kw | 640,972 |  |
|  | #2 | clinical outcome*':ti,ab,kw OR 'critical care outcome*':ti,ab,kw OR 'patient-reported outcome*':ti,ab,kw | 415,041 |  |
|  | #3 | older adult*':ti,ab,kw OR 'over 65*':ti,ab,kw OR 'elderly*':ti,ab,kw OR 'geriatric*':ti,ab,kw OR 'over 80*':ti,ab,kw OR ‘aged*’:ti,ab,kw OR 'aged'/exp OR 'very elderly'/exp | 4,530,680 |  |
|  | **#4** | **#1 AND #2 AND #3 AND [01-12-2021]/sd NOT [01-03-2023]/sd** | **414** |  |
| **PubMed** | #1 | (((((((((((((((((machine learning* [Title/Abstract]) OR (artificial intelligence*[Title/Abstract])) OR (deep learning*[Title/Abstract])) OR (statistical learning*[Title/Abstract])) OR (algorithm*[Title/Abstract])) OR (expert system*[Title/Abstract])) OR (neural network*[Title/Abstract])) OR (natural language processing*[Title/Abstract])) OR (predictive model*[Title/Abstract])) OR (machine learning*[Text Word])) OR (artificial intelligence*[Text Word])) OR (deep learning*[Text Word])) OR (statistical learning*[Text Word])) OR (algorithm*[Text Word])) OR (expert system*[Text Word])) OR (neural network*[Text Word])) OR (natural language processing*[Text Word])) OR (predictive model*[Text Word]) | 678,926 |  |
|  | #2 | ((((((clinical outcome*[Title/Abstract]) OR (critical care outcome*[Title/Abstract])) OR (patient-reported outcome*[Title/Abstract])) OR (clinical outcome[Text Word])) OR (clinical outcome*[Text Word])) OR (critical care outcome*[Text Word])) OR (patient-reported outcome*[Text Word]) | 263,858 |  |
|  | #3 | (((((((((((((older adult*[Title/Abstract]) OR (over 65*[Title/Abstract])) OR (elderly*[Title/Abstract])) OR (geriatric*[Title/Abstract])) OR (over 80*[Title/Abstract])) OR (aged*[Title/Abstract])) OR (very elderly*[Title/Abstract])) OR (older adult*[Text Word])) OR (over 65*[Text Word])) OR (elderly*[Text Word])) OR (geriatric*[Text Word])) OR (over 80*[Text Word])) OR (aged*[Text Word])) OR (very elderly*[Text Word]) | 6,053,672 |  |
|  | **#4** | **#1 AND #2 AND #3 Filters: from 2021/12/1 - 2023/2/28** | **109** |  |
| **Web of Science Core collection** | #1 | TS = ("machine learning*" or "artificial intelligence*" or "deep learning*" or "statistical learning*" or "algorithm*" or "expert system*" or "neural network*" or "natural language processing*" or "predictive model*") | 3,324,223 |  |
|  | #2 | TS = ("clinical outcome*" or "critical care outcome* " or "patient-reported outcome*") | 292,183 |  |
|  | #3 | TS = ("older adult*" or "over 65*" or "elderly*" or "geriatric*" or "over 80*" or "aged*" or "very elderly*") | 1,237,508 |  |
|  | **#4** | **#1 AND #2 AND #3 and 2023 or 2022 or 2021 (Publication Years)** | **89** |  |
| **Web of Science BIOSIS citation index** | #1 | TS = ("machine learning*" or "artificial intelligence*" or "deep learning*" or "statistical learning*" or "algorithm*" or "expert system*" or "neural network*" or "natural language processing*" or "predictive model*") | 337,621 |  |
|  | #2 | TS = ("clinical outcome*" or "critical care outcome* " or "patient-reported outcome*") | 137,777 |  |
|  | #3 | TS = ("older adult*" or "over 65*" or "elderly*" or "geriatric*" or "over 80*" or "aged*" or "very elderly*") | 1,677,302 |  |
|  | **#4** | **#1 AND #2 AND #3 and 2022 or 2023 (Publication Years)** | **112** |  |
| **Scopus** | #1 | ( TITLE-ABS-KEY ( "machine learning" ) OR TITLE-ABS-KEY ( "artificial intelligence*" ) OR TITLE-ABS-KEY ( "deep learning*" ) OR TITLE-ABS-KEY ( "statistical learning" ) OR TITLE-ABS-KEY ( "algorithm*" ) OR TITLE-ABS-KEY ( "expert system*" ) OR TITLE-ABS-KEY ( "neural network*" ) OR TITLE-ABS-KEY ( "natural language processing*" ) OR TITLE-ABS-KEY ( "predictive model*" ) ) | 4,928,389 |  |
|  |  |  |  |  |
|  | #2 | ( TITLE-ABS-KEY ( "clinical outcome*" ) OR TITLE-ABS-KEY ( "critical care outcome*" ) OR TITLE-ABS-KEY ( "patient reported outcome*" ) ) | 434,883 |  |
|  | #3 | ( TITLE-ABS-KEY ( "older adult*" ) OR TITLE-ABS-KEY ( "over 65*" ) OR TITLE-ABS-KEY ( "over 80*" ) OR TITLE-ABS-KEY ( "aged*" ) OR TITLE-ABS-KEY ( "elderly*" ) OR TITLE-ABS-KEY ( "very elderly*" ) OR TITLE-ABS-KEY ( "geriatric*" ) ) | 6,325,412 |  |
|  |  |  |  |  |
|  | **#4** | **#1 AND #2 AND #3 AND ( LIMIT-TO ( PUBYEAR , 2023 ) OR LIMIT-TO ( PUBYEAR , 2022 ) )** | **3,511** |  |
| **ProQuest** | #1 | ab(“machine learning*” OR “artificial intelligence*” OR “deep learning*” OR “statistical learning*” OR “algorithm*” or “expert system*” OR “neural network*” OR “natural language processing*” OR “predictive model*”) OR ti(“machine learning*” OR “artificial intelligence*” OR “deep learning*” OR “statistical learning*” OR “algorithm*” or “expert system*” OR “neural network*” OR “natural language processing*” OR “predictive model*”) | 885,835 |  |
|  | #2 | ab(“clinical outcome*” OR “critical care outcome*” OR “patient reported outcome*”) OR ti(“clinical outcome*” OR “critical care outcome*” OR “patient reported outcome*”) | 44,453 |  |
|  | #3 | ab(“older adult*” OR “over 65*” OR “elderly*” OR “geriatric*” OR “over 80*” OR “aged*” OR “very elderly*”) OR ti(“older adult*” OR “over 65*” OR “elderly*” OR “geriatric*” OR “over 80*” OR “aged*” OR “very elderly*”) | 365,276 |  |
|  | **#4** | **#1 AND #2 AND #3 AND** Limits Applied (Date of publication 2022, 2023.) | **18** |  |

**Supplementary Information Table 2.** *PROBAST assessment.*

| **Authors; Year; Country** | **Participants** | **Predictors** | **Outcomes** | **Analysis** | **Overall Risk of Bias** |
| --- | --- | --- | --- | --- | --- |
| **Abdul Ghffar et al; 2020; USA** |  |  |  |  |  |
| OptiML fusion model predicting in-hospital cardiovascular mortality. | Low | Low | Low | Low | Low |
| OptiML fusion model predicting in-hospital all-cause mortality. | Low | Low | Low | Low | Low |
| OptiML fusion model predicting 30-day cardiovascular mortality. | Low | Low | Low | Low | Low |
| OptiML fusion model predicting 30-day all-cause mortality. | Low | Low | Low | Low | Low |
| **Belmin et al, 2022; France** |  |  |  |  |  |
| Random Forest model predicting 14-day risk of ED admission. | Low | Low | Low | Low | Low |
| **Bories et al, 2022 ; France** |  |  |  |  |  |
| RF model predicting hospitalisation for bleeding events. | Low | Low | Low | Low | Low |
| SVM model predicting hospitalisation for bleeding events. | Low | Low | Low | Low | Low |
| XGBoost model predicting hospitalisation for bleeding events. | Low | Low | Low | Low | Low |
| **Bowen et al; 2021; USA** |  |  |  |  |  |
| Multilevel mixed modelling predicting pain perception in the last 7 days. | Low | Low | Unclear | Unclear | Unclear |
| **Chen et al; 2020; China** |  |  |  |  |  |
| Subdistribution hazards regression model predicting survival in patients with early stage uterine papillary carcinoma. | Low | Low | Low | Low | Low |
| **Chung et al; 2020; Taiwan** |  |  |  |  |  |
| ANN model 1 predicting favourable or poor outcome at 3 months following AIS. | Low | Low | Low | Unclear | Unclear |
| ANN model 2 predicting favourable or poor outcome at 3 months following AIS. | Low | Low | Low | Unclear | Unclear |
| **Considine et al; 2019; Australia** |  |  |  |  |  |
| Multivariable logistic regression model predicting emergency interhospital transfer from subacute to acute care. | Low | Low | Low | Low | Low |
| **Das et al; 2003; USA** |  |  |  |  |  |
| ANN model predicting recurrent bleeding. | Low | Low | Low | Low | Low |
| ANN model predicting death. | Low | Low | Low | Low | Low |
| ANN model predicting therapeutic interventions for control of haemorrhage. | Low | Low | Low | Low | Low |
| Multiple-logistic regression model predicting recurrent bleeding. | Low | Low | Low | Low | Low |
| Multiple-logistic regression model predicting death. | Low | Low | Low | Low | Low |
| Multiple-logistic regression model predicting therapeutic interventions for control of haemorrhage. | Low | Low | Low | Low | Low |
| **Diaz-Ramirez et al; 2021; USA** |  |  |  |  |  |
| baBIC model predicting time to first ADL dependence. | Low | Low | Low | Low | Low |
| baBIC model predicting time to first IADL difficulty. | Low | Low | Low | Low | Low |
| baBIC model predicting time to first mobility dependence. | Low | Low | Low | Low | Low |
| baBIC model predicting time to death. | Low | Low | Low | Low | Low |
| **Duarte et al; 2015; USA** |  |  |  |  |  |
| PROMPT model predicting time to death. | Low | Low | Low | Low | Low |
| **Falsetti et al; 2021; Italy** |  |  |  |  |  |
| XGBoost model predicting therapeutic failure. | Low | Low | Low | Low | Low |
| XGBoost model predicting stroke/TIA. | Low | Low | Low | Low | Low |
| XGBoost model predicting major bleeding. | Low | Low | Low | Low | Low |
| **Ford et al; 2021; UK** |  |  |  |  |  |
| Logistic Regression model predicting dementia. | Low | Low | Low | Low | Low |
| Naïve Bayes model predicting dementia. | Low | Low | Low | Low | Low |
| RF model predicting dementia. | Low | Low | Low | Low | Low |
| **Fransvea et al, 2022 ; Italy** |  |  |  |  |  |
| Elastic-Net model predicting 30-day-mortality. | Low | Low | Low | Low | Low |
| SVM model predicting 30-day-mortality. | Low | Low | Low | Low | Low |
| KNN model predicting 30-day-mortality. | Low | Low | Low | Low | Low |
| DT Classifier model predicting 30-day-mortality. | Low | Low | Low | Low | Low |
| Multilayer Perceptron model predicting 30-day-mortality. | Low | Low | Low | Low | Low |
| **Friz et al, 2022 ; Italy** |  |  |  |  |  |
| Adaptive Boosting model predicting 30-day all-cause readmissions after decompensated heart failure. | Low | Low | Low | Low | Low |
| Gradient Boosting model predicting 30-day all-cause readmissions after decompensated heart failure. | Low | Low | Low | Low | Low |
| XGBoost model predicting 30-day all-cause readmissions after decompensated heart failure. | Low | Low | Low | Low | Low |
| Random Forest model predicting 30-day all-cause readmissions after decompensated heart failure. | Low | Low | Low | Low | Low |
| **Gomes et al; 2021; Germany** |  |  |  |  |  |
| ANN model predicting all-cause intrahospital mortality. | Low | Low | Low | Low | Low |
| RF model predicting all-cause intrahospital mortality. | Low | Low | Low | Low | Low |
| SVM model predicting all-cause intrahospital mortality. | Low | Low | Low | Low | Low |
| **Han et al; 2012; USA** |  |  |  |  |  |
| Logistic regression model predicting six-month mortality. | Low | Low | Low | Low | Low |
| **Ko et al; 2014; USA** |  |  |  |  |  |
| Linear discriminant 10-variable model predicting colonoscopy average risk screening. | Low | Low | Low | Low | Low |
| Linear discriminant 30-variable model predicting colonoscopy average risk screening. | Low | Low | Low | Low | Low |
| CART 7 variable model predicting colonoscopy average risk screening. | Low | Low | Low | Low | Low |
| Linear discriminant 10-variable model predicting colonoscopy high risk screening. | Low | Low | Low | Low | Low |
| Linear discriminant 30-variable model predicting colonoscopy high risk screening. | Low | Low | Low | Low | Low |
| CART 7 variable model predicting colonoscopy high risk screening. | Low | Low | Low | Low | Low |
| Linear discriminant 10-variable model predicting colonoscopy surveillance. | Low | Low | Low | Low | Low |
| Linear discriminant 30-variable model predicting colonoscopy surveillance. | Low | Low | Low | Low | Low |
| CART 7 variable model predicting colonoscopy surveillance. | Low | Low | Low | Low | Low |
| Linear discriminant 10-variable model predicting colonoscopy diagnostic. | Low | Low | Low | Low | Low |
| Linear discriminant 30-variable model predicting colonoscopy diagnostic. | Low | Low | Low | Low | Low |
| CART 7 variable model predicting colonoscopy diagnostic. | Low | Low | Low | Low | Low |
| **Li Kuan Ong et al, 2023 ; Sigapore, UK, Australia** |  |  |  |  |  |
| Model 1 predicting dose accumulated, Grade 1 GU toxicity. | Low | Low | Low | Low | Low |
| Model 1a predicting dose predicted, Grade 1 GU toxicity. | Low | Low | Low | Low | Low |
| Model 2 predicting Grade 2 GU toxicity. | Low | Low | Low | Low | Low |
| **Maurer et al, 2023 ; USA** |  |  |  |  |  |
| POTTER model predicting 30-day-mortality. | Low | Low | Low | Low | Low |
| **Morris et al; 2020; USA** |  |  |  |  |  |
| fEMAT model predicting all-cause in-hospital mortality. | Low | Low | Low | Low | Low |
| qEMAT model predicting all-cause in-hospital mortality. | Low | Low | Low | Low | Low |
| **Ocagli et al; 2021; Italy** |  |  |  |  |  |
| RF model predicting 4AT delirium score. | Low | Low | Low | Unclear | Unclear |
| **Parenica et al; 2012; Czech Republic** |  |  |  |  |  |
| EuroSCORE model predicting 1-year mortality . | Low | Low | Low | Unclear | Unclear |
| **Pilotto et al; 2010; Italy** |  |  |  |  |  |
| MPI model predicting 30-day-mortality . | Low | Low | Low | Low | Low |
| NYHA model predicting 30-day-mortality . | Low | Low | Low | Low | Low |
| EFFECT model predicting 30-day-mortality . | Low | Low | Low | Low | Low |
| ADHERE model predicting 30-day-mortality . | Low | Low | Low | Low | Low |
| **Pompei et al; 1994; USA** |  |  |  |  |  |
| Logistic regression model predicting Delirium. | Low | Low | Low | Low | Low |
| **Ren et al, 2022 ; China** |  |  |  |  |  |
| RF model predicting the occurrence of in-hospital complications within 30 days of admission. | Low | Low | Low | Unclear | Unclear |
| **Rossi et al; 2021; Italy** |  |  |  |  |  |
| Multivariable Cox model predicting the probabilities of developing myeloid neoplasms. | Low | Low | Low | Low | Low |
| **Sancarlo et al; 2012; Italy** |  |  |  |  |  |
| MPI model predicting 1-month all-cause mortality. | Low | Low | Low | Low | Low |
| MPI model predicting 6-month all-cause mortality. | Low | Low | Low | Low | Low |
| MPI model predicting 12-month all-cause mortality. | Low | Low | Low | Low | Low |
| **Sax et al; 2021; USA** |  |  |  |  |  |
| Logistic Regression model predicting any 30-day serious adverse event. | Low | Low | Low | Low | Low |
| LASSO model predicting any 30-day serious adverse event. | Low | Low | Low | Low | Low |
| DT model predicting any 30-day serious adverse event. | Low | Low | Low | Low | Low |
| RF model predicting any 30-day serious adverse event. | Low | Low | Low | Low | Low |
| XGBoost model predicting any 30-day serious adverse event. | Low | Low | Low | Low | Low |
| **Shardell et al; 2021; USA** |  |  |  |  |  |
| Weighted DT model predicting sex-specific serum 25-hydroxyvitamin D thresholds that best discriminated incident slow gait. | Low | Low | Low | Low | Low |
| **Suzuki et al; 2020; Japan** |  |  |  |  |  |
| Multiple logistic regression model predicting 180-day all-cause mortality. | Low | Low | Low | Low | Low |
| **Thongprayoon et al, 2023 ; USA, Thailand** |  |  |  |  |  |
| Unsupervised consensus clustering model predicting distinct clusters of patients and their post-transplant outcomes. | Low | Low | Low | Low | Low |
| **Velagapudi et al; 2021; USA** |  |  |  |  |  |
| Logistic regression model predicting thrombolysis in cerebral infarction on first pass. | Low | Low | Low | Low | Low |
| RF model predicting thrombolysis in cerebral infarction on first pass. | Low | Low | Low | Low | Low |
| SVM model predicting thrombolysis in cerebral infarction on first pass. | Low | Low | Low | Low | Low |
| Naïve Bayes model predicting thrombolysis in cerebral infarction on first pass. | Low | Low | Low | Low | Low |
| XGBoost model predicting thrombolysis in cerebral infarction on first pass. | Low | Low | Low | Low | Low |
| **Venerito et al, 2022 ; Italy** |  |  |  |  |  |
| Logistic regression model predicting 3-month giant cell arthritis flare. | Low | Low | Low | Low | Low |
| Simple DT model predicting 3-month giant cell arthritis flare. | Low | Low | Low | Low | Low |
| RF model predicting 3-month giant cell arthritis flare. | Low | Low | Low | Low | Low |
| **Venturini et al; 2021; Italy** |  |  |  |  |  |
| Conditional RF (M1) predicting discharge. | Low | Low | Low | Low | Low |
| RF (M2) predicting discharge. | Low | Low | Low | Low | Low |
| Ordinal forest (M3) predicting discharge. | Low | Low | Low | Low | Low |
| Partitional tree (M4) predicting discharge. | Low | Low | Low | Low | Low |
| Conditional inference tree (M5) predicting discharge. | Low | Low | Low | Low | Low |
| Conditional RF (M1) predicting decease (mortality). | Low | Low | Low | Low | Low |
| RF (M2) predicting decease (mortality). | Low | Low | Low | Low | Low |
| Ordinal forest (M3) predicting decease (mortality). | Low | Low | Low | Low | Low |
| Partitional tree (M4) predicting decease (mortality). | Low | Low | Low | Low | Low |
| Conditional inference tree (M5) predicting decease (mortality). | Low | Low | Low | Low | Low |
| Conditional RF (M1) predicting ICU transfer. | Low | Low | Low | Low | Low |
| RF (M2) predicting ICU transfer. | Low | Low | Low | Low | Low |
| Ordinal forest (M3) predicting ICU transfer. | Low | Low | Low | Low | Low |
| Partitional tree (M4) predicting ICU transfer. | Low | Low | Low | Low | Low |
| Conditional inference tree (M5) predicting ICU transfer. | Low | Low | Low | Low | Low |
| **Verdu-Rotellar et al, 2022 ; Spain** |  |  |  |  |  |
| Multivariable logistic regression predicting 30-day mortality. | Low | Low | Low | Low | Low |
| Multivariable logistic regression predicting 30-day hospitalisation. | Low | Low | Low | Low | Low |
| **Zarkowsky et al; 2021; USA** |  |  |  |  |  |
| Multivariable logistic regression model predicting short-stay discharge appropriateness. | Low | Low | Low | Low | Low |
| Multivariable logistic regression model predicting survival at 30 days. | Low | Low | Low | Low | Low |
| Multilayer perceptron model predicting short-stay discharge appropriateness. | Low | Low | Low | Low | Low |
| Multilayer perceptron model predicting survival at 30 days. | Low | Low | Low | Low | Low |
| **Zhou et al; 2021; USA** |  |  |  |  |  |
| GA-KPLS model predicting heart failure with preserved ejection fraction risk. | Low | Low | Low | Low | Low |
| LASSO model predicting heart failure with preserved ejection fraction risk. | Low | Low | Low | Low | Low |
| RF model predicting heart failure with preserved ejection fraction risk. | Low | Low | Low | Low | Low |
| Ridge Regression model predicting heart failure with preserved ejection fraction risk. | Low | Low | Low | Low | Low |
| SVM model predicting heart failure with preserved ejection fraction risk. | Low | Low | Low | Low | Low |
| Logistic Regression model predicting heart failure with preserved ejection fraction risk. | Low | Low | Low | Low | Low |

**Supplementary Information Appendix 3.** *PRISMA checklist.*


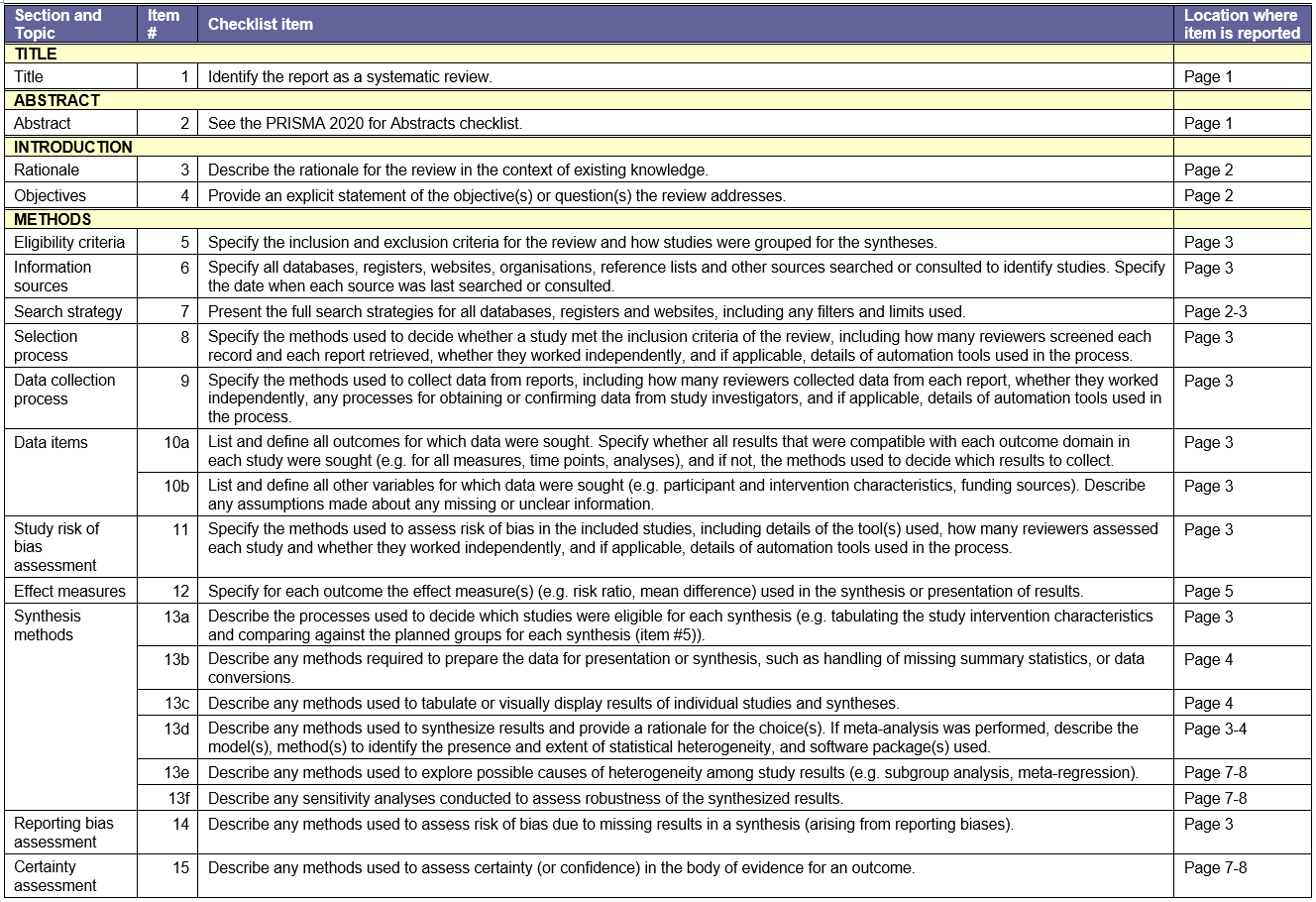


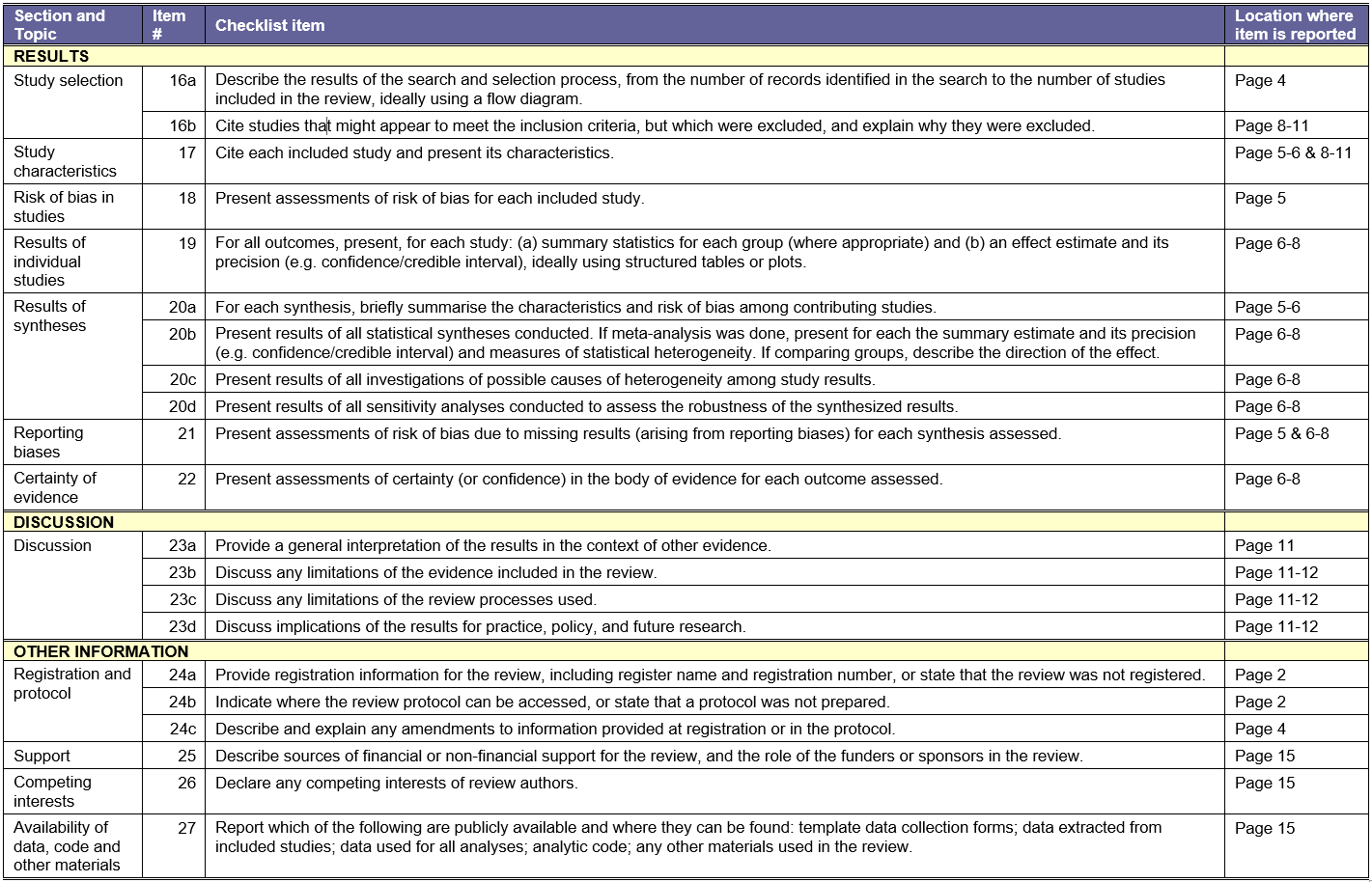


**References**

1. Team J. JASP (Version 0.16.3)[Computer software]. JASP Team; 2022.
